# The Association of Childhood Maltreatment With Lipid Peroxidation and DNA Damage in Postpartum Women

**DOI:** 10.3389/fpsyt.2019.00023

**Published:** 2019-02-18

**Authors:** Christina Boeck, Anja M. Gumpp, Alexandra M. Koenig, Peter Radermacher, Alexander Karabatsiakis, Iris-Tatjana Kolassa

**Affiliations:** ^1^Clinical and Biological Psychology, Institute of Psychology and Education, Ulm University, Ulm, Germany; ^2^Institute of Anesthesiological Pathophysiology and Process Engineering, University Hospital Ulm, Ulm, Germany

**Keywords:** childhood maltreatment, oxidative stress, lipid peroxidation, DNA damage, 8-isoprostane, 8-OH(d)G, comet assay, γH2AX

## Abstract

Childhood maltreatment (CM) is associated with an increased risk for the development of psychiatric and somatic disorders in later life. A potential link could be oxidative stress, which is defined as the imbalance between the amount of reactive oxygen species (ROS) and the neutralizing capacity of anti-oxidative defense systems. However, the findings linking CM with oxidative stress have been inconsistent so far. In this study, we aimed to further explore this association by investigating biological markers of DNA and lipid damage due to oxidation in a comprehensive approach over two study cohorts of postpartum women (study cohort I and study cohort II). The severity of CM experiences (maltreatment load) was assessed in both studies using the *Childhood Trauma Questionnaire*. In study cohort I (*N* = 30), we investigated whether CM was associated with higher levels of structural DNA damage in peripheral blood mononuclear cells (PBMC) by two methods that are highly sensitive for detecting nuclear DNA strand breaks (comet assay and γH2AX staining). In study cohort II (*N* = 117), we then assessed in a larger cohort, that was specifically controlled for potential confounders for oxidative stress measurements, two established serum and plasma biomarkers of oxidative stress, one representing oxidative DNA and RNA damage (8-hydroxy-2′-deoxyguanosine and 8-hydroxyguanosine; 8-OH(d)G) and the other representing lipid peroxidation (8-isoprostane). In study cohort I, the analyses revealed no significant main effects of maltreatment load on cellular measures of nuclear DNA damage. The analyses of peripheral oxidative stress biomarkers in study cohort II revealed a significant main effect of maltreatment load on free 8-isoprostane plasma levels, but not on total 8-isprostane plasma levels and 8-OH(d)G serum levels. Taken together, by combining different methods and two study cohorts, we found no indications for higher oxidative DNA damages with higher maltreatment load in postpartum women. Further research is needed to investigate whether this increase in free 8-isoprostane is a marker for oxidative stress or whether it is instead functionally involved in ROS-related signaling pathways that potentially regulate inflammatory processes following a history of CM.

## Introduction

The experience of emotional, physical and/or sexual abuse, as well as emotional and/or physical neglect during childhood (i.e., childhood maltreatment [CM]) may cast a long shadow on adult health: A growing body of literature has shown that individuals with a history of CM are at greater risk to develop both mental and physical disorders later in life (1). Although accumulating research supports the hypothesis that CM is biologically embedded and exerts a long-lasting influence on stress-responsive systems (2, 3), research elucidating the causal pathways underlying the association of CM with adult health outcomes is nevertheless sparse. The research investigating this potential link focuses more and more on oxidative stress, which is defined as the imbalance between the amount of reactive oxygen species (ROS) and the neutralizing capacity of anti-oxidative defense systems (4).

Physiologically, ROS are produced in several subcellular structures (mainly within mitochondria) and serve important signaling functions that are essential for the coordination of metabolic, inflammatory, and stress response-related processes (4, 5). If the amount of ROS production, however, exceeds the physiological level and cannot be counterbalanced by the body's antioxidant defense systems, ROS readily attack lipids, proteins, DNA, and RNA (6). These ROS-induced modifications are also often applied in biomedical research as stable biomarkers to assess states of oxidative stress. One of the most investigated biomarkers for oxidative stress is 8-isoprostane, a specific peroxidation product of arachidonic acid and therefore a marker of lipid peroxidation (7). The levels of the oxidized nucleobase guanine within DNA (8-hydroxy-2′-deoxyguanosine; 8-OHdG) and RNA (8-hydroxyguanosine; 8-OHG) are often used as circulating markers for oxidative DNA and RNA damage (8). In addition, (oxidative) DNA damage can further be assessed on a cellular level by the comet assay or by the staining for phosphorylated histone H2AX (γH2AX). While the comet assay is a direct measure for DNA single and double strand breaks (9), γH2AX plays a role in signaling DNA double strand breaks and initiating their repair by supporting the recruitment and localization of DNA repair proteins (10). If such oxidative damages accumulate over time, they may have detrimental effects both at the cellular and at the systemic level (6, 11).

So far, oxidative stress was found to be involved in many physical diseases, like migraine, neurodegenerative diseases, cardiovascular diseases, and cancer (12–15)—amongst them several disorders that are observed at higher rates in individuals with a history of CM (1). Oxidative stress and related damages have also been implicated more and more in psychiatric disorders (16), including depression [see (17) for a review], bipolar disorder [see (18) for a meta-analysis], schizophrenia (19, 20), posttraumatic stress disorder (PTSD) (21), anxiety disorders (22), and different personality disorders (23). In affective disorders, higher oxidative stress and decreased antioxidant enzyme activities were associated with a lower health-related quality of life (24). Not only psychiatric disorders, but also other psychological stress factors like psychosocial stress (25), subjectively perceived stress (26, 27), chronic caregiving stress (27, 28), intimate partner violence (29), and sociodemographic disadvantage (30) were all reported to be associated with increased oxidative stress levels.

With regard to CM, findings have however been inconsistent so far: While do Prado et al. (31) reported that CM was associated with higher plasma levels of oxidative-stress-related protein carbonylation and an imbalance between oxidative molecules and antioxidants, Fanning et al. (23) found no significant association between CM and plasma levels of oxidative stress biomarkers (8-OH(d)G and 8-isoprostane) in individuals with different personality disorders. Additionally, Bergholz et al. (32) recently showed an association between complex childhood traumatization and nuclear DNA damage (γH2AX staining) in peripheral blood lymphocytes, while Simsek et al. (33) previously reported that children with a history of childhood sexual abuse did not differ in serum levels of antioxidant enzymes, the antioxidant coenzyme Q, and DNA damage (8-OH(d)G) from children without such experiences.

By investigating risk and resilience factors in the transgenerational transmission of CM in two study cohorts of postpartum women (study cohort I and study cohort II), we also found evidence for alterations in serum oxidative stress biomarkers and serum antioxidants applying targeted (study cohort I) and untargeted (study cohort II) metabolomics analyses (34, 35). Study cohort I showed reduced serum levels of metabolites with antioxidant capacity (L-carnitine and acetylcarnitine) and increased biomarkers of oxidative stress (Arginine-to-Citrulline ratio) (34). In study cohort II, untargeted metabolomics indicated higher serum levels of bilirubin IXa, another metabolite with antioxidant capacity, among women with CM compared to non-exposed women (35). Bilirubin is an end product of heme degradation by heme oxygenase-1 (HO-1), an enzyme with known anti-inflammatory and anti-oxidative properties (36, 37). Accordingly, higher levels of serum bilirubin were previously suggested to reflect the intensity of initial oxidative stress (38). Further analyses in study cohort I investigating the respiratory activity of mitochondria—the main producers of ROS—in intact peripheral blood mononuclear cells (PBMC), showed that CM was not only associated with alterations in mitochondrial activity, but also indicated an increase in cellular ROS production with increasing severity of CM experiences (34). These measures were further associated with a pro-inflammatory status of PBMC as represented by an increased spontaneous release of pro-inflammatory cytokines (34). As mitochondria and ROS are critical regulators of inflammatory processes (11, 39, 40), these findings suggest that alterations in mitochondrial activity and ROS production might not only constitute stress-related cellular damages but could also be functionally involved in adaptive signaling pathways. In the same study cohort, we observed that telomeres, the protective caps of our chromosomes that are more vulnerable for oxidative DNA damages than the rest of the genome (41), were significantly shorter in the long-living immune cell subset of memory cytotoxic T cells in women with CM compared to those without (42).

In sum, the complex picture arising on CM-related changes in ROS levels and the question whether oxidative stress is of physiological importance with regard to its signaling function or has damaging effects, remains far from being understood. Continuing our previous analyses in study cohort I (34) and study cohort II (35), this study aimed to investigate markers of oxidative DNA and lipid damage in a comprehensive approach over these two study cohorts of postpartum women with CM. In study cohort I (*N* = 30), we investigated whether CM was associated with higher levels of oxidative DNA damage in PBMC by two methods that are highly sensitive for detecting nuclear DNA strand breaks (comet assay and γH2AX staining). In study cohort II (*N* = 117), we then assessed in this larger, independent study cohort, that was specifically controlled for potential confounders for oxidative stress measurements, two established blood serum and plasma biomarkers of oxidative stress, one representing oxidative DNA and RNA damage (8-hydroxy-2′-deoxyguanosine and 8-hydroxyguanosine; 8-OH(d)G) and the other representing lipid peroxidation (8-isoprostane).

## Materials and Methods

### Design and Procedure of Study Cohort I and Study Cohort II

Participants of two longitudinal studies (study cohort I and study cohort II; see Measures in study cohort I and Analyses in study cohort II for detailed description), both investigating risk and resilience factors in the transgenerational transmission of CM, were used for the analyses. Study cohort I constituted thereby the pilot study to show the feasibility for a large-scale assessment, i.e., study cohort II, which was part of the project “My Childhood—Your Childhood.” For both studies, women were recruited shortly after giving birth to a child (< 1 week postpartum) at the maternity ward of the Ulm University Hospital (time point t_0_). Exclusion criteria for study participation were maternal age under 18 years, severe health problems of mother or child, severe complications during parturition, and an insufficient knowledge of the German language. Participating mother-infant-dyads were then accompanied over 1 year with two follow-up assessments, the first 3 months postpartum (t_1_) and the second 12 months postpartum (t_2_). The studies were approved by the Ethics Committee of Ulm University and all procedures followed the current version of the Declaration of Helsinki (43).

After providing written informed consent, women were retrospectively interviewed about their history of maltreatment experiences below the age of 18 years with the German short version of the *Childhood Trauma Questionnaire* (44–46). The CTQ covers the five CM subscales emotional, physical, and sexual abuse as well as emotional and physical neglect. The CTQ sum score (range 25–125) was used as a cumulative measure for the severity of maltreatment experiences, the so-called *maltreatment load* (47). Using standardized cut-off criteria for the classification of CM based on CTQ sum scores (44, 45), participants were categorized into “no CM,” “low CM,” “moderate CM,” and “severe CM” based on reported CM experiences for recruitment, follow-up, and selection of study participants for biological analyses (see Study participants of study cohort I and Study participants of study cohort II). In addition to the assessment of CM experiences, women were further asked to provide basic socio-demographic information at t_0_.

During the follow-up interview at t_1_, women provided detailed socio-demographic, clinical, and medical data in self-report. Additionally, whole blood samples were collected by venipuncture between 11 a.m. and 2:30 p.m. for the isolation of PBMC, plasma, and serum samples (EDTA-Monovettes for plasma collection and for whole blood sampling for PBMC isolation as well as S-Monovettes for serum collection; Sarstedt, Nümbrecht, Germany). To minimize additional acute psychological strain, the study participants were not obligated to fast overnight prior to the assessment.

#### Serum C-Reactive Protein (CRP) Content

To exclude participants who presented with signs of an acute inflammatory status at t_1_, we assessed the serum CRP levels in all participants of study cohort I and study cohort II. For serum collection, whole blood was centrifuged for 10 min at 3,000 g and 4°C. Serum samples were aliquoted and stored frozen at −80°C until further analysis. Afterwards, serum CRP levels were measured at the Central Facility for Clinical Chemistry of the University Hospital Ulm using a chemiluminescence immunoassay analyzed on a Cobas 6,000 platform (Roche Diagnostics, Risch, Switzerland) for study cohort I and on a Cobas 8,000 platform (Roche Diagnostics, Risch, Switzerland) for study cohort II. One participant of study cohort I and three participants of study cohort II showed a CRP level >10 mg/l, which is indicative of an acute infection, and were therefore excluded from all subsequent analyses.

### Measures in Study Cohort I

#### Study Participants of Study Cohort I

In study I (conducted from March 2012–May 2013), a total of 240 women gave written informed consent and participated in the screening interview (t_0_). Oversampling for individuals with a higher maltreatment load, 112 women were invited and 67 actually participated at the follow-up interview 3 months postpartum (t_1_; see Supplementary Figure S1 for detailed description of study flow and drop-out rates). Applying the established cut-off criteria of the CTQ (44, 45), 25 of these women were categorized as having no CM experiences, 22 as having low CM experiences, five as having moderate CM experiences, and 15 as having severe CM experiences. As study participants with moderate and severe CM experiences were significantly younger than women with no or low CM experiences [*F*_(3, 47)_ = 3.76, *p* = 0.017], a subsample of 31 participants was selected out of this total study cohort to match women with no and low CM experiences and women with moderate and severe CM experiences for age [see (34) for a detailed description]. Body mass index (BMI) was a secondary matching criterion as BMI influences levels of oxidative stress (48, 49). The final study cohort selected for biological analyses consisted of *N* = 8 women with no, *N* = 8 women with low, *N* = 4 women with moderate and *N* = 11 women with severe CM experiences. Additionally, one study participant with low CM experiences was subsequently excluded from the analyses, as the serum CRP level indicated the presence of an acute inflammatory status [see Serum C-reactive protein (CRP) content]. Thus, reported statistical data of study cohort I are based on a final sample of *N* = 30 women (see Supplementary Figure S2 for the distribution of the maltreatment load).

#### Isolation of Peripheral Blood Mononuclear Cells (PBMC)

For the assessment of nuclear DNA damage, PBMC were isolated from whole blood by Ficoll-Hypaque gradient centrifugation according to the manufacturer's protocol (GE Healthcare, Chalfont St. Giles, UK) immediately after blood sampling. Isolated cells were stored at −80°C in cryopreservation medium (dimethyl sulphoxide: Sigma-Aldrich, St. Louis, MO, USA; fetal calf serum: Sigma-Aldrich; dilution 1:10). For the analyses, frozen PBMC were thawed, washed twice in phosphate-buffered saline (PBS) at room temperature and counted with trypan blue staining for the quantification of living cells. An aliquot of 1 × 10^5^ cells was then used for the detection of nuclear strand breaks by comet assay and an aliquot of 5 × 10^5^ cells was fixated in a 3:1 (v/v) solution of methanol (Sigma-Aldrich) and glacial acetic acid (VWR, Radnor, PA VWR, Radnor, PA, USA) for the detection of γH2AX foci.

#### Comet Assay

The comet assay measures DNA strand breaks (single strand and double strand breaks) after lysis of the cells (9). The alkaline version of the comet assay (single-cell gel electrophoresis) was performed on PBMC as previously described by Speit and Hartmann (50). In short, 5 × 10^4^ cells were suspended in an agarose gel on a microscopy slide. Following lysis (for at least 1 h), cells were denatured with alkali (pH 13) for 30 min and electrophoresis was performed for 25 min at 25 V and 300 mA using a Consort Electronics power supply ev231 (CONSORT, Turnhout, Belgium). Slides were subsequently stained with ethidium bromide (Roth, Karlsruhe, Germany) for the analysis of the DNA migration distance by fluorescence microscopy (Olympus BX41 U-LH100HG, Olympus, Tokyo, Japan; Supplementary Figure S3). The software Comet Assay II (Perceptive Instruments, Haverhill, UK) was used to determine the median tail intensity (percentage of DNA in the tail) and median tail moment (tail intensity × tail length) of 100 randomly selected cells per slide on two slides per sample. For each run, a positive control (x-ray irradiated Hela cells) and a negative control (non-irradiated Hela cells) were included. The measures tail intensity and tail moment were used for statistical analyses.

#### Detection of γH2AX Foci

As a marker for DNA double strand breaks (10), we measured phosphorylated histone H2AX (γH2AX) in intact cells. For fluorescence staining, 1 × 10^5^ fixated cells were spread out onto superfrost slides (Menzel-Glaeser, Braunschweig, Germany) and washed with PBS (2 × 5 min). Subsequently, cells were permeabilized with pepsin for 10 min at 37°C, washed twice in washing buffer (70% [v/v] formamide, 10 mM Tris base, 0.1% [w/v] bovine serum albumin) for 20 min each, twice in TBS-Tween (1%) for 5 min each, and twice in PBS for 5 min each. All cover slips were then treated with 200 μl primary antibody solution (Anti-phospho-Histone H2A.X [Ser139], Merck, Millipore, Billerica, MA, USA) diluted 1:1,000 in blocking buffer (0.9 M PBS, 19% [w/v] bovine serum albumin, 0.1% v/v Tween 20) and incubated over night at 4°C. On the next day, the slides were washed with PBS (2 × 5 min) and then incubated with 200 μl secondary antibody solution (goat anti-mouse Alexa-Fluor 488 nm, Life Technologies, Carlsbad, CA, USA; dilution 1:300 in blocking buffer) for 1 h in a humid chamber at room temperature. Thereafter, the slides were washed with PBS (2 × 5 min). Finally, cell nuclei were counterstained with DAPI using Vectashield Mounting Medium (Vector Laboratories, Burlingame, CA, USA). Analysis of γH2AX was performed using a Leica DM5000 B fluorescent microscope (Leica Microsystems, Wetzlar, Germany), and images were taken at a 1,000-fold magnification and an exposure time of 400 ms (Supplementary Figure S4). One hundred cells per sample were assessed and the number of γH2AX foci was counted manually. In each run, a positive control (x-ray irradiated Hela cells) and a negative control (non-irradiated Hela cells) were included. For statistical analyses, the following two measures were applied: the number of γH2AX foci per cell (γH2AX foci/cell) and the percentage of cells with γH2AX foci.

### Analyses in Study Cohort II

#### Study Participants of Study Cohort II

The participants of study cohort II were recruited within the “My Childhood—Your Childhood” project which was conducted from October 2013 to December 2016. After providing written informed consent, 533 women participated at t_0_ in study II [see (51) for a detailed description]. Three months postpartum, 285 of these women participated at t_1_ (see Supplementary Figure S5 for detailed description of study flow and drop-out rates) in a detailed psychodiagnostic interview. Lifetime psychiatric disorders were diagnosed by trained psychologists with the German version of the *Structured Clinical Interview* (SCID-I) (52) for the diagnosis of major axis I disorders of the *Diagnostic and Statistical Manual of Mental Disorders* [4th ed., text rev.; DSM-IV-TR; (53)].

At t_1_, we were able to obtain peripheral blood samples from 252 women for the generation of serum and plasma samples. According to the established cut-off criteria of the CTQ (44, 45), 141 of these women were categorized as having no CM experiences, 52 as having low CM experiences, 28 as having moderate CM experiences, and 31 as having severe CM experiences. In order to validate the association between the maltreatment load and oxidative stress biomarkers in a sample that was controlled for potential confounding factors known to influence oxidative stress biomarkers such as current cigarette smoking (54) and BMI (48, 49), we excluded women who reported current smoking at t_1_ (*N* = 14: *N*_noCM_ = 6, *N*_lowCM_ = 3, *N*_moderateCM_ = 2, and *N*_severeCM_ = 3) and women with a BMI > 30 kg/m^2^; *N* = 22: *N*_noCM_ = 10, *N*_lowCM_ = 3, *N*_moderateCM_ = 4, and *N*_severeCM_ = 5) from the biological analyses in study cohort II. Furthermore, women with autoimmune diseases (*N* = 12: *N*_noCM_ = 7, *N*_lowCM_ = 2, *N*_moderateCM_ = 2, and *N*_severeCM_ = 1), non-Caucasian ethnicity (*N*_noCM_ = 1), acute intake of psychotropic medication (*N*_severeCM_ = 1), acute illness (self-report; *N* = 24: *N*_noCM_ = 14, *N*_lowCM_ = 5, *N*_moderateCM_ = 3, and *N*_severeCM_ = 2), and missing psychological data (*N*_lowCM_ = 1) were excluded. For women without CM experiences, a lifetime history of a psychiatric disorder (*N* = 28) and experiences of severe distress within the last 3 months (e.g., death of a close person; *N* = 10) were applied as further exclusion criteria. From the remaining *N* = 65 women without CM experiences, *N* = 46 were selected for oxidative stress analysis due to limited capacity of financial resources. Additionally, three study participants (two women with no CM experiences and one with severe CM experiences) were excluded from the statistical analyses as the serum CRP levels indicated the presence of an acute inflammatory status [see Serum C-reactive protein (CRP) content]. To this end, the final study cohort II (*N* = 117) consisted of 44 women with no CM experiences, 38 women with low CM experiences, 17 women with moderate CM experiences and 18 women with severe CM experiences. CM experiences (see Supplementary Figure S6 for the distribution of the maltreatment load).

#### Blood Sampling

In study cohort II, oxidative stress parameters were assessed in serum and plasma samples. Therefore, whole blood (one pre-chilled S-Monovette for serum and one pre-chilled EDTA-Monovette for plasma sampling), was centrifuged for 10 min at 3,000 g and 4°C. Serum and plasma samples were aliquoted and stored frozen at −80°C until further analysis. Serum samples were used for the quantification of 8-OH(d)G and plasma samples for the assessment of free and total 8-isoprostane levels.

#### Oxidative Stress Parameters in Serum and Plasma

Serum 8-OH(d)G levels were quantified using the *DNA/RNA Oxidative Damage ELISA Kit* (Item No. 589320, Cayman Chemical, Ann Arbor, MI, USA) according to the manufacturer's protocol. This immunoassay covers three oxidized guanine species as marker for DNA/RNA oxidative damage: 8-hydroxy-2′-deoxaguanosine from DNA, 8-hydroxyguanosine from RNA and 8-hydroxyguanine from either DNA or RNA. As recommended in the manufacturer's protocol, serum samples were diluted 1:25 prior to analysis. The assay has a range from 10.3 to 3,000 pg/ml and a sensitivity of approximately 30 pg/ml. As markers for lipid peroxidation, free (circulating) and total 8-isoprostane levels were measured in blood plasma. Total 8-isoprostane is a combination of free 8-isoprostane and 8-isoprostane that is esterified to phospholipids. Free and total plasma 8-isoprostane levels were measured using the *8-isoprostane ELISA Kit* (Item No. 516351, Cayman Chemical, Ann Arbor, MI, USA) according to the manufacturer's protocol. The assay has a range from 0.8 to 500 pg/ml and a sensitivity of approximately 3 pg/ml. For the measurement of free 8-isoprostane, plasma samples were used untreated, whereas an additional alkaline hydrolysis step was performed for the analysis of total 8-isoprostane. Analyses were performed in thawed samples in duplicates and averaged values were used for statistical analyses. Samples were randomly distributed over the plates to prevent any batch effects.

### Statistical Analyses

All biological analyses were performed blinded with respect to clinical variables. Statistical analyses were performed using R version 3.5.0 (55) and *p*-values < 0.05 were considered as significant. In accordance with the findings that the risk for developing PTSD after traumatic experiences increases with increasing traumatic load (56, 57), there is accumulating evidence pointing toward a dose-response-relationship between the severity of maltreatment experiences (maltreatment load) and associated biological alterations (34, 51, 58, 59). Therefore, we tested for an association between cellular, serum, and plasma biomarkers of oxidative stress-related damages and the CTQ sum score as a continuous measure for the maltreatment load. Due to skewness, non-normality, and outliers in oxidative stress measures as well as in the maltreatment load, the use of traditional parametric methods was inappropriate. Thus, the nonparametric probabilistic index model (PIM) of Thas et al. (60), a robust rank-based equivalent of the generalized linear model, was applied [R package “pim” version 2.0.0.2: (61)]. Due to the relatively small sample size, no covariates were included in the statistical analyses in study cohort I, which was, however, matched for age and BMI to minimize the influence of these potential confounders. For study cohort II, the influence of potential confounders for oxidative stress measurements (smoking, obesity, autoimmune diseases, non-Caucasian ethnicity, acute intake of psychotropic medication, and acute illness) was minimized using exclusion criteria (see Study participants of study cohort II). Age was included as covariate in the statistical analyses of study cohort II as oxidative stress was found to be involved in aging (62). The probability (P) for an increase of the outcome variable was modeled as a function of the predictors (study cohort I: maltreatment load; study cohort II: maltreatment load and age). The estimates (*b*), 95% confidence intervals (CI) of the estimates (*b*[95% CI]), standard errors (SE *b*), as well as related *z*-statistics and *p*-values were used for these rank-based regression models.

## Results: Childhood Maltreatment and Oxidative Stress Biomarkers in Study Cohort I and Study Cohort II

All descriptive sociodemographic and biological data of study cohort I and study cohort II can be found in Tables 1 and 2, respectively. The levels of humoral oxidative stress markers (8-OH(d)G, free 8-isoprostane, and total 8-isoprostane) in maternal blood at t_1_ did not differ significantly between the different types of delivery at t_0_ (all *p* > 0.18). For a graphical overview of the biological raw data see Supplementary Figure S7. All results of the probabilistic index models reported in the following are summarized in Table 3. With regard to cellular measures of (oxidative) DNA damage assessed in study cohort I, the analyses revealed no significant main effects of maltreatment load—as measured by the CTQ sum score—on tail intensity (*b* = −0.0011, *p* = 0.95) and tail moment (*b* = −0.0035, *p* = 0.83). These results were confirmed by γH2AX fluorescence staining, with no significant main effects of maltreatment load on γH2AX foci/cell (*b* = 0.0065, *p* = 0.68) and the percentage of cells with γH2AX foci (*b* = −0.0004, *p* = 0.97).

**Table 1:** Socio-demographic and clinical data of study cohort I and study cohort II.

	**Study cohort I**		**Study cohort II**	
	**(*****N*** **= 30)**		**(*****N*** **= 117)**	
	**Mean ± SD or *N* (%)**	**Range**	**Mean ± SD or *N* (%)**	**Range**
**DEMOGRAPHICS**				
Age (years)	31.6 ± 6.0	22 – 44	33.0 ± 4.1	23 – 43
BMI (kg/m^2^)	25.3 ± 6.4	19 – 47	23.7 ± 3.0^a^	16.5 – 30
Smoking status [yes, *N* (%)]	8 (28.6 %)^b^	–	–	–
Ethnicity [Caucasian, *N* (%)]	29 (96.7 %)^c^	–	117 (100 %)	–
Number of children	2 ± 1	1 – 5	2 ± 1^a^	1 – 4
Living in a partnership [yes, *N* (%)]	30 (100 %)		114 (99.1 %)^d^	–
Academic education [yes, *N* (%)]	13 (43.3 %)	–	88 (75.9 %)^a^	–
Vaginal delivery [yes, *N* (%)]	30 (100 %)	–	89 (76.7 %)^a^	–
Time interval between last food intake and blood drawing (minutes)	125.5 ± 125.9^e^	5 – 480	114.7 ± 76.1^a^	0 – 333
**ADVERSITY AND PSYCHIATRIC SYMPTOM LOAD**				
CTQ sum score	42.8 ± 14.2	25 – 73	35.0 ± 10.0	25 – 81
Emotional abuse sum score	9.8 ± 5.3	5 – 21	7.4 ± 3.4	5 – 21
Physical abuse sum score	7.1 ± 3.8	5 – 18	6.0 ± 2.5	5 – 21
Sexual abuse sum score	6.7 ± 4.1	5 – 25	5.9 ± 3.1	5 – 21
Emotional neglect sum score	12.3 ± 4.8	5 – 22	9.9 ± 3.9	5 – 18
Physical neglect sum score	6.9 ± 2.9	5 – 16	5.9 ± 1.8	5 – 15
**Psychiatric diagnoses lifetime**	**Self-report**		**SCID diagnosis**^a^	
Depressive disorder [*N* (%)]	6 (20.0 %)	–	15 (12.9 %)	–
Anxiety disorder [*N* (%)]	2 (6.7 %)	–	21 (18.1 %)^f^	–
Borderline personality disorder [*N* (%)]	2 (6.7 %)^g^	–	–	–
Eating disorder [*N* (%)]	1 (3.3 %)^h^	–	–	–
Compulsive disorder [*N* (%)]	1 (3.3 %)	–	1 (0.9 %)	–
Alcohol use disorder [*N* (%)]	–	–	4 (3.4 %)	–
Stimulant use disorder [*N* (%)]	–	–	2 (1.7 %)	–
Trauma–related disorders [*N* (%)]	–	–	4 (3.4 %)	–
**CHRONIC ILLNESSES^i^**	**Self-report**		**Self-report**^**a**^	
Thyroid disease [*N* (%)]	5 (16.7 %)	–	22 (19.0 %)	–
Hypertension [*N* (%)]	2 (6.7 %)	–	–	–
Allergy [*N* (%)]	1 (3.3 %)	–	2 (1.7 %)	–
Asthma [*N* (%)]			7 (6.0 %)	–
Epilepsy [*N* (%)]	–	–	2 (1.7 %)	–
Neurodermatitis [*N* (%)]	–	–	2 (1.7 %)	–
**MEDICATION^j^**	**Self-report**		**Self-report**^**a**^	
L-Thyroxin [*N* (%)]	4 (13.3 %)	–	26 (22.4 %)	–
Psychotropic medication [*N* (%)]	3 (10.0 %)	–	–	–
Oral contraceptives [*N* (%)]	1 (3.3 %)	–	14 (12.1 %)	–
Analgesic [*N* (%)]	–	–	6 (5.2 %)	–
Asthma inhaler [*N* (%)]	–	–	2 (1.7 %)	–

*BMI, Body mass index; CTQ, Childhood Trauma Questionnaire; SCID, Structured Clinical Interview*.

a *N = 116, one missing value*.

b *N = 28, two missing values*.

c *One study participant of Brazilian origin*.

d *N = 115, two missing values*.

e *N = 27, three missing values*.

f *One subject with depressive disorder, anxiety disorder and trauma-related disorder*.

g *One subject with lifetime Borderline personality disorder and anxiety disorder*.

h *One subject with lifetime diagnosis of eating disorder and mild depression*.

i *Chronic illnesses that have been reported by more than two study participants. In study cohort I, one study participant reported each the following chronic illnesses: asthma, chronic bronchitis, colitis ulcerosa, epilepsy, and psoriasis vulgaris. In study cohort II, one study participant reported each the following chronic illnesses: chronic venous insufficiency, circular hair loss, coagulation disorder, hay fever, prediabetes, prolaktinoma, protein S deficiency, prothrombin mutation, Scheuermann's disease, scoliosis, and von Willebrand disease*.

j *Medication reported if at least 2 study participants reported intake*.

**Table 2:** Cellular, serum, and plasma measures of oxidative stress biomarkers in study cohort I and study cohort II.

	**Mean ± SD**	**Range**	**Median**	**IQR**
**STUDY COHORT I (*N*=30)**				
Tail Intensity (%)	2.97 ± 1.64	0.74–8.52	2.60	1.75
Tail Moment (AU)	0.26 ± 0.16	0.08–0.90	0.24	0.16
γH2AX foci/cell^a^	0.31 ± 0.28	0.01–1.03	0.22	0.30
% cells with γH2AX foci^a^	18.97 ± 14.23	1–50	16	20
**STUDY COHORT II (*N* = 117)**				
8-OH(d)G levels (pg/ml)	4456 ± 1076	1336–7480	4343	1571
Free 8-isoprostane levels (pg/ml)	36.8 ± 79.5	2.1–538.2	20.1	11.9
Total 8-isoprostane levels (pg/ml)	364.7 ± 187.5	97.9–1094.2	323.0	254.5

a *N = 29*.

**Table 3:** Probabilistic Index Model results on the association between the CTQ sum score and cellular (study cohort I), serum, and plasma measures (study cohort II) of oxidative stress-related damage.

**Regressor**	**b**	**b [95% CI]**	**SE**	**z**	***p***
**STUDY COHORT I (*****N*** **= 30)**					
Tail Intensity	−0.0011	[−0.0333;0.0312]	0.02	−0.06	0.95
Tail Moment	−0.0035	[−0.0348;0.0279]	0.02	−0.22	0.83
γH2AX foci/cell^a^	0.0065	[−0.0249;0.0380]	0.02	0.41	0.68
% cells with γH2AX foci^a^	−0.0004	[−0.0298;0.0288]	0.02	−0.03	0.97
**STUDY COHORT II (*N* = 117)^b^**					
8-OH(d)G	0.0155	[−0.0076;0.0386]	0.01	1.32	0.19
Free 8-isoprostane	0.0277	[0.0065;0.0490]	0.01	2.56	0.01
Total 8-isoprostane	0.0187	[−0.0043;0.0417]	0.01	1.60	0.11

*CI, Confidence interval; CTQ, Childhood Trauma Questionnaire*.

a *N = 29*.

b *Probabilistic Index Models in study cohort II included maternal age as covariate*.

**Figure 1:**
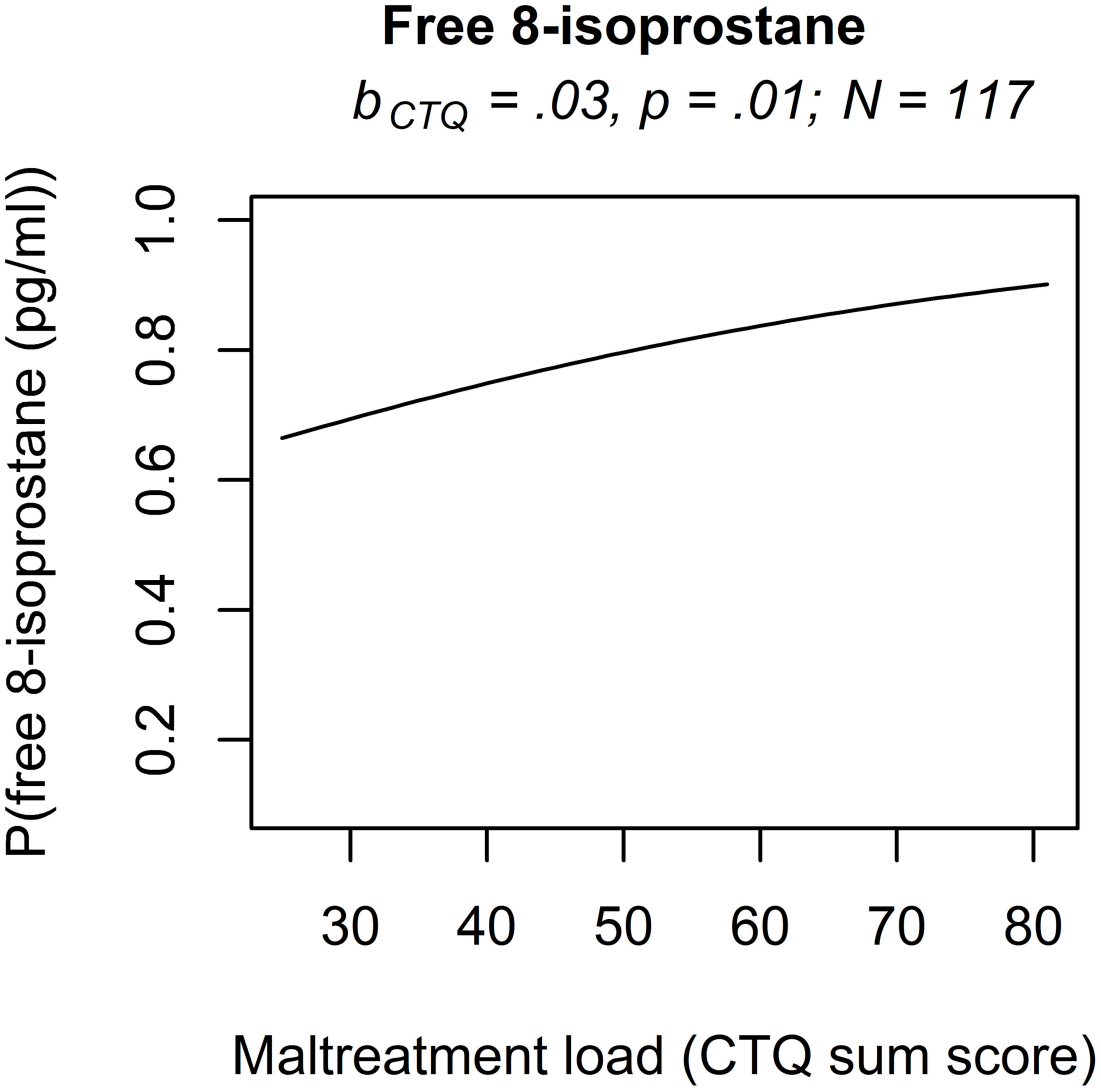
Effect for the maltreatment load (CTQ sum score) on free 8-isoprostane in study cohort II. The results of the rank-based regression model for free 8-isoprostane are visualized as the probability (P) for an increase of free 8-isoprostane modeled as a function of the predictors (maltreatment load (CTQ sum score) and age as covariate). Free 8-isoprostane was measured as marker for lipid peroxidation in study cohort II (*N* = 117). b_CTQ_, Estimate for the main effect of the CTQ sum score; CTQ, *Childhood Trauma Questionnaire*; P, Probability for an increase of the outcome variable modeled as a function of the predictors.

The analyses of serum and plasma oxidative stress biomarkers assessed in study cohort II revealed a significant main effect of maltreatment load on free 8-isoprostane levels (*b* = 0.0277, *p* = 0.01), but not on total 8-isprostane (*b* = 0.0187, *p* = 0.11) and 8-OH(d)G (*b* = 0.0155, *p* = 0.19) levels. Thus, the probability for higher free 8-isoprostane levels increased significantly with a higher maltreatment load (Figure 1). No significant main effects were found for the covariate age on free and total 8-isoprostane and 8-OH(d)G levels, respectively (*p* > 0.05). Results remained the same when one outlier in total 8-isoprostane (1094.2 pg/ml) and four outliers in free 8-isoprostane (>200 pg/ml) were excluded from the respective analyses. Including the time interval between the last food intake and blood drawing as additional covariate in our statistical analyses of study cohort I and study cohort II did not alter the significance of the results.

## Discussion

We comprehensively assessed serum, plasma, and cellular measures that are well-established biomarkers of oxidative stress-related damages—i.e., DNA/RNA damages and lipid peroxidation—over two study cohorts of postpartum women with a history of childhood maltreatment. In both cohorts, we previously found indications for oxidative imbalances in relation to CM: in study cohort I we showed that CM was associated with increased ROS production and reduced levels of L-carnitine and acetylcarnitine, two serum metabolites that inherit antioxidant capacities (34), while untargeted metabolomics analyses revealed a higher signal intensity for bilirubin in CM-affected individuals of study cohort II (35). Bilirubin is an end product of heme degradation by HO-1. The enzyme itself as well as its degradation products, like bilirubin, were reported to have immunomodulatory, anti-inflammatory, and anti-oxidative properties (36–38). Building on these results, the analyses of two cellular measures of nuclear DNA damage (DNA migration in the comet assay and the appearance of γH2AX foci within the cell nucleus) indicated now, however, that CM and in particular the maltreatment load was not related to an increase in oxidative DNA damage in PBMC in study cohort I. This finding was further supported by the analyses conducted in study cohort II, a cohort controlled for the potential confounding factors smoking and BMI, where we did neither find a significant association between maltreatment load and the serum levels of 8-OH(d)G. Thus, by combining three different methods and two study cohorts, we consistently found that CM was not related to oxidative DNA damages in postpartum women. With regard to lipid peroxidation, we found that an increasing maltreatment load was significantly associated with a higher probability for increased plasma levels of free 8-isoprostane, but not with plasma levels of total 8-isoprostane in study cohort II. In line with previous findings from our group (34, 51, 58, 59), we found with regard to free 8-isoprostane level again a significant influence of the severity of CM experiences, thus supporting the hypothesis of a dose-dependent effect of maltreatment load. Together, these results bear several potential suggestions: (1) that an increase in ROS levels and associated oxidation products in CM-affected individuals might not only be seen with regard to its damaging potential, but might instead serve a functional role, (2) that CM-related oxidative damages may be persistent at the level of lipid peroxidation, while DNA repair mechanisms may counterbalance and thus cope with oxidative stress-induced DNA damages, and (3) that exogenous or endogenous resilience factors may influence the association of CM with oxidative stress-related damages. These potential implications will be discussed in the following.

Consistent with our findings, two previous studies did not report any significant associations between childhood abuse and neglect and serum 8-OH(d)G levels in children (33) and in adults with and without different personality disorders (23). Fanning et al. (23) further found in the same cohort no association between CM and plasma 8-isoprostane levels, but the authors did not specify whether they assessed free or total 8-isoprostane. While we found no significant alterations in total 8-isoprostane levels, we found a significantly increased probability of higher free 8-isoprostane plasma levels with higher maltreatment load in postpartum women. In contrast to esterified 8-isoprostane that constitutes the major fraction of total 8-isoprostane levels, free 8-isoprostane can not only be generated by the non-enzymatic, ROS-mediated peroxidation of arachidonic acid, but also by an alternate enzymatic pathway that is catalyzed by the inflammation-induced *prostaglandin-endoperoxidase synthase* (63–66). It was therefore recently suggested that an increase in total 8-isoprostane may be indicative of oxidative stress, whereas a sole increase in free 8-isoprostane may rather point to the involvement of inflammatory processes (15). For the interpretation of 8-isoprostane results, it is therefore necessary to always consider both pathways, the oxidative stress-related and the inflammation-related pathway. With regard to these two pathways, Eick et al. (30) previously reported a higher chemical fraction of 8-isoprostane urine levels in pregnant women with poor psychosocial status (e.g., high anxiety levels, high depression levels, low self-esteem, low mastery, and high subjective stress), but no difference in the enzymatic fraction by investigating the ratio of 8-isoprostane to prostaglandin F2α. These results point toward the presence of increased oxidative stress levels in association with psychosocial disadvantages as well as with extremely stressful life events, such as family death, during pregnancy (30). In contrast, we found a significant association between the severity of maltreatment experiences in childhood and free 8-isoprostane levels, but not total 8-isoprostane levels. Thus, our findings in study cohort II might be indicative of increased chronic inflammatory processes associated with CM in postpartum women rather than of increased oxidative stress. CM has been consistently associated with a phenotype of chronic low-grade inflammation [reviewed in (67)]. In line with this suggestion, we previously found in study cohort I not only that CM was associated with increased immuno-cellular ROS production in postpartum women, but also that this increase in ROS production was further associated with a pro-inflammatory status of the cells (34). Indeed, mounting evidence indicates that ROS are not only by-products of mitochondrial oxidative phosphorylation, but also have important signaling functions and are involved in pathways regulating anti-microbial effects (68), apoptosis (69), autophagy (70), and inflammation (39, 40). Excessive ROS production by mitochondria can drive the gene-expression and production of pro-inflammatory cytokines through activation of pro-inflammatory transcription factors (e.g., NFκB) and through activation of the NLRP3 inflammasome (39, 40). Subsequently, inflammation can also induce ROS production by inflammatory cells leading to higher levels of oxidative stress (5). Thus, the observation of increased ROS production and ROS-related oxidation products with CM could be a sign of inflammatory signaling processes rather than for high oxidative stress levels causing cellular damage.

A second potential explanation for the observed difference in oxidative DNA/RNA and lipid damages with CM might lie in a difference in repair, metabolism, and excretion dynamics. In contrast to lipids, DNA repair mechanism may counterbalance and thus cope with oxidative stress-induced DNA damages (71, 72). As such, lipid peroxidation might persist and constitute a long-term marker of stress experiences, while oxidative DNA damages might rather be observable in association with acute stress experiences or it might take more severe levels of psychological and oxidative stress to induce persistent oxidative DNA damages. Consistent with this hypothesis, it was recently reported that adult psychiatric patients with a history of complex childhood traumatization presented significantly higher levels of nuclear DNA damage in PBMC as assessed by γH2AX staining compared to healthy individuals and also compared to psychiatric patients with low levels of complex childhood traumatization (32). Complex childhood traumatization was here defined as the experience of sexual, physical or emotional abuse by a primary caregiver or another member of the family or social group the victim belongs to (32). Investigating refugees with a high traumatic load, we also reported that individuals with PTSD showed higher levels of basal DNA breakage in PBMC compared to trauma-exposed subjects without PTSD and non-trauma-exposed control subjects (21). Individuals with PTSD showed, however, a higher cellular capacity to repair single-strand breaks after exposure to ionizing x-radiation (21), which may point toward a trauma-specific effect on cellular DNA repair processes. Cellular repair mechanisms of oxidative DNA damages may play an even stronger role in protecting the DNA against mutations, which can—if they are not recognized and repaired—lead to a higher risk for somatic diseases like cancer (73).

Although CM constitutes a major risk factor for both adverse mental and physical disorders, not all individuals with a history of CM develop pathological health outcomes in the long-term. It therefore has been suggested that individual vulnerability and resilience factors such as the genetic background, but also environmental, behavioral, and psychosocial factors can influence “how deep CM gets under the skin” (74, 75). In line with this suggestion, our working group showed that women with CM reported lower levels of perceived stress, if they concomitantly reported higher levels of social support (76). We further showed that the stress-related hormone cortisol potentiates the effect of CM on telomere length shortening and on the increase in immune-cellular oxygen consumption (42, 59). On the other hand, the attachment-related hormone oxytocin may buffer the biological effects of childhood maltreatment on telomere length and cellular oxygen consumption (42, 59). Furthermore, there is first evidence that nutrition like the supplementation with omega-3 fatty acid has beneficial effects on lipid peroxidation (77). Future studies should therefore take genetic, psychosocial, behavioral, and biological factors into account to further dissect the association of CM with oxidative stress states and related cellular and structural damages.

While the present study has several strengths such as the consistency of the observed findings across different research methods across two study cohorts, there are also some limitations: Biological assessment in our study was conducted three months postpartum. The postpartum period is characterized by major life transitions, which are particularly stressful for mothers with a history of CM (76). Therefore, increased current perceived stress and adverse childhood experiences are comorbid and it is difficult to disentangle the effects on oxidative stress markers. By investigating this specific study cohort, we were able to analyze differences in oxidative stress markers with respect to negative childhood maltreatment experiences in a highly demanding and sensitive time period.

Pregnancy and parturition are not only associated with social and psychological alterations, but also with biological alterations characterized by substantial changes in the maternal immune and endocrine system (78, 79). Pregnancy and especially the third trimester is furthermore associated with increased oxidative stress markers in women with uncomplicated pregnancies (80). However, it was further reported that most of the oxidative stress markers had returned to non-pregnant levels 6 to 8 weeks postpartum and were comparable to those of non-pregnant and non-postpartum women (80). Furthermore, our reported oxidative stress values are comparable to those of non-pregnant women (15, 81). According to these findings, it can be assumed that pregnancy-related changes in the oxidative stress system had mostly normalized at the time point of biological assessment in our studies. Nevertheless, the results need to be replicated in non-postpartum women and investigated also in men to show the generalizability of the findings.

Our study cohorts consisted of healthy, non-clinical community samples with a relatively high socioeconomic status. As the socioeconomic status is a protective factor for mental health (82), the high socioeconomic status of our study cohorts might contribute to the observation of small effects of the maltreatment load on oxidative stress markers in blood.

Due to ethical considerations, we collected non-fasting blood samples for mothers who were potentially still breastfeeding their children three months postpartum. Non-fasting blood collection could also have an effect on the oxidative stress markers measured in blood, which should be analyzed in future studies. We comprehensively assessed serum, plasma, and cellular measures that are well-established and stable biomarkers of oxidative stress-related damages. However, they are all indirect markers for oxidative stress. Future studies should use new technologies, for example electro-spin-resonance (ESR), to directly measure ROS in blood and biological fluids (83).

Furthermore, the intake of medication, mainly of thyroid hormones, as well as the presence of comorbid diseases, both somatic and psychiatric disorders, might have an effect on the measured oxidative stress levels. Exclusion of individuals with somatic or psychiatric disorders would lead to a non-representative study cohort as negative health outcomes are observed at higher rates in CM-affected individuals (1). Nevertheless, the influence of different co-morbid chronic and psychiatric disorders in individuals with a history of CM on oxidative stress parameters has to be investigated in more detail in further studies.

## Conclusion

In conclusion, a history of CM was associated with higher plasma levels of free 8-isoprostane, but not with total 8-isoprostane in postpartum women. By combining different methods and two study cohorts, we found no indications for higher oxidative DNA damages with higher maltreatment load in postpartum women. Further research is needed to investigate whether the increase in free 8-isoprostane is a persistent marker for oxidative stress or whether it is instead functionally involved in ROS-related signaling pathways that potentially regulate inflammatory processes following a history of CM. Additionally, even in non-psychiatric cohorts with CM, possible treatment effects by behavioral, psychotherapeutic, or mental stress coping interventions should be investigated for their protective potential against the biological sequelae of early life adversities to reduce the risk for mental as well as physical health conditions in the aftermath of CM.

## Author Contributions

Study cohort I was part of a pilot project to show the feasibility of study II. Study cohort II was part of the project “My Childhood—Your Childhood,” funded by the Federal Ministry of Education and Research of Germany between 2013 and 2016. Both projects were, among others, conceptualized by I-TK, and AKa. For both studies, I-TK provided additional funding for the biological analyses and supervised all stages of the project. AKo recruited the women, performed the screening as well as diagnostic interviews, collected and preprocessed clinical data. CB and AG conducted the biological analyses. PR contributed analytical tools. CB and AKo performed statistical analyses and interpreted the data together with AG, AKa, and I-TK. AG and CB wrote the manuscript with input and critical revisions from all authors. All authors approved the final manuscript.

### Conflict of Interest Statement

The authors declare that the research was conducted in the absence of any commercial or financial relationships that could be construed as a potential conflict of interest.

## Abbreviations

8-OHdG: 8-hydroxy-2'-deoxyguanosine
8-OHG: 8-hydroxyguanosine
BMI: body mass index
CI: confidence interval
CM: childhood maltreatment
CRP: C-reactive protein
CTQ: *Childhood Trauma Questionnaire*
HO-1: heme oxygenase-1
PBMC: peripheral blood mononuclear cells
PIM: probabilistic index model
PTSD: posttraumatic stress disorder
ROS: reactive oxygen species
SCID: Structured Clinical Interview
γH2AX: phosphorylated histone H2AX.
