# Supplementary material for: The Association of Childhood Maltreatment With Lipid Peroxidation and DNA Damage in Postpartum Women
Source: Front Psychiatry. 2019 Feb 18;10:23. doi: 10.3389/fpsyt.2019.00023 (PMC6387959; doi:10.3389/fpsyt.2019.00023)
Supplement: Supplementary file 1 [file Data_Sheet_1.pdf]

frontiers

Supplementary Material

The association of childhood maltreatment with lipid peroxidation and DNA damage in postpartum women

Christina Boeck[1,3], Anja M. Gumpp[1,3*], Alexandra M. Koenig[1], Peter Radermacher[2], Alexander Karabatsiakis[1], Iris-Tatjana Kolassa[1]

[1]Clinical & Biological Psychology, Institute of Psychology and Education, Ulm University, Ulm, Germany.

[2]Institute of Anesthesiological Pathophysiology and Process Engineering, University Hospital Ulm, Ulm, Germany.

[3]Shared first-authors.

*** Correspondence:** Anja M. Gumpp: anja.gumpp@uni-ulm.de

1 Supplementary Figures

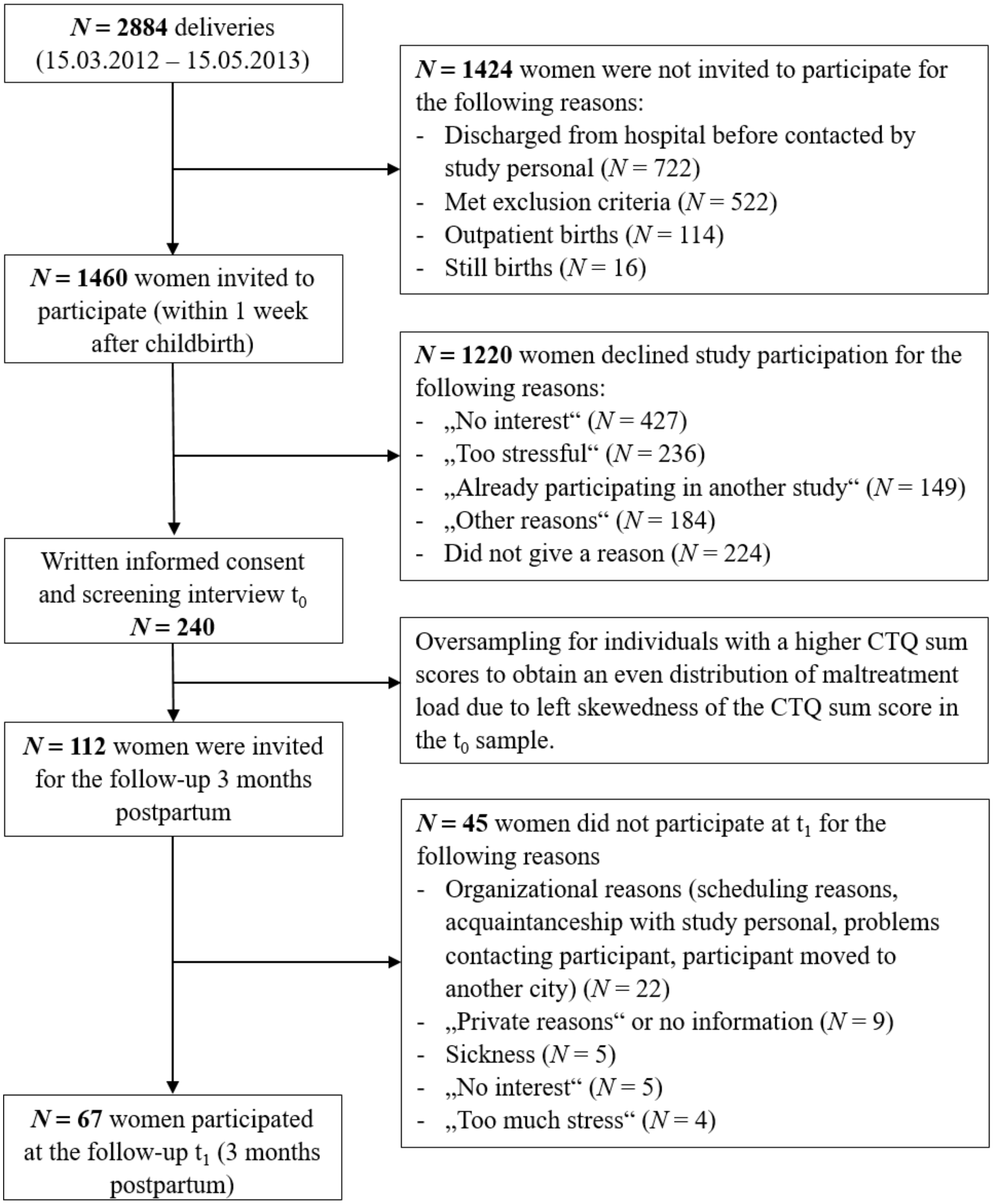

Supplementary Figure S.1: Study design of study cohort I with drop-out rates.

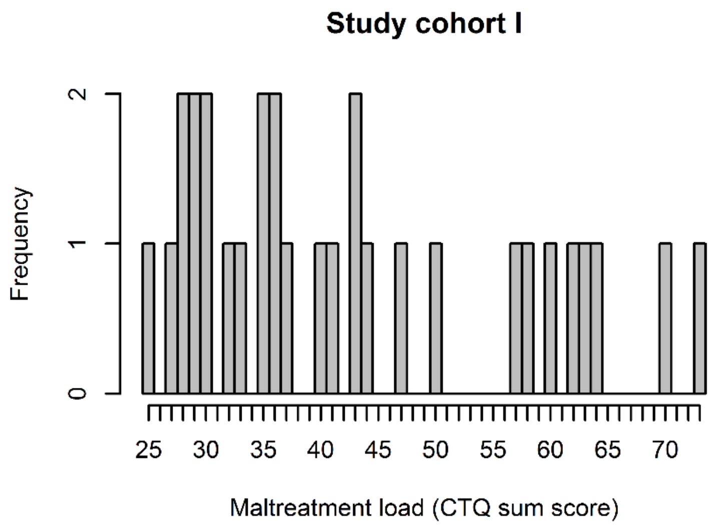

Supplementary Figure S.2: Distribution of the maltreatment load in study cohort I (*N* = 30).
CTQ = *Childhood Trauma Questionnaire*.

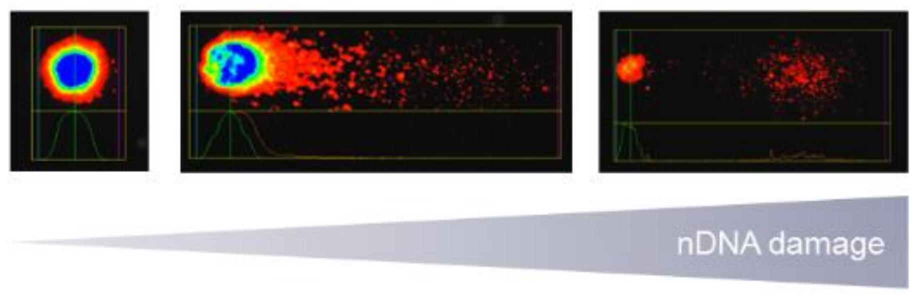

Supplementary Figure S.3: Exemplary representation of the Comet Assay showing different levels of nuclear DNA (nDNA) damage in PBMC represented by the DNA migration distance in the agarose gel matrix. Left: undamaged (no tail DNA); middle and right: increasing levels of DNA damage (increasing amount of nDNA in the tail).
nDNA = nuclear DNA; PBMC = peripheral blood mononuclear cells.

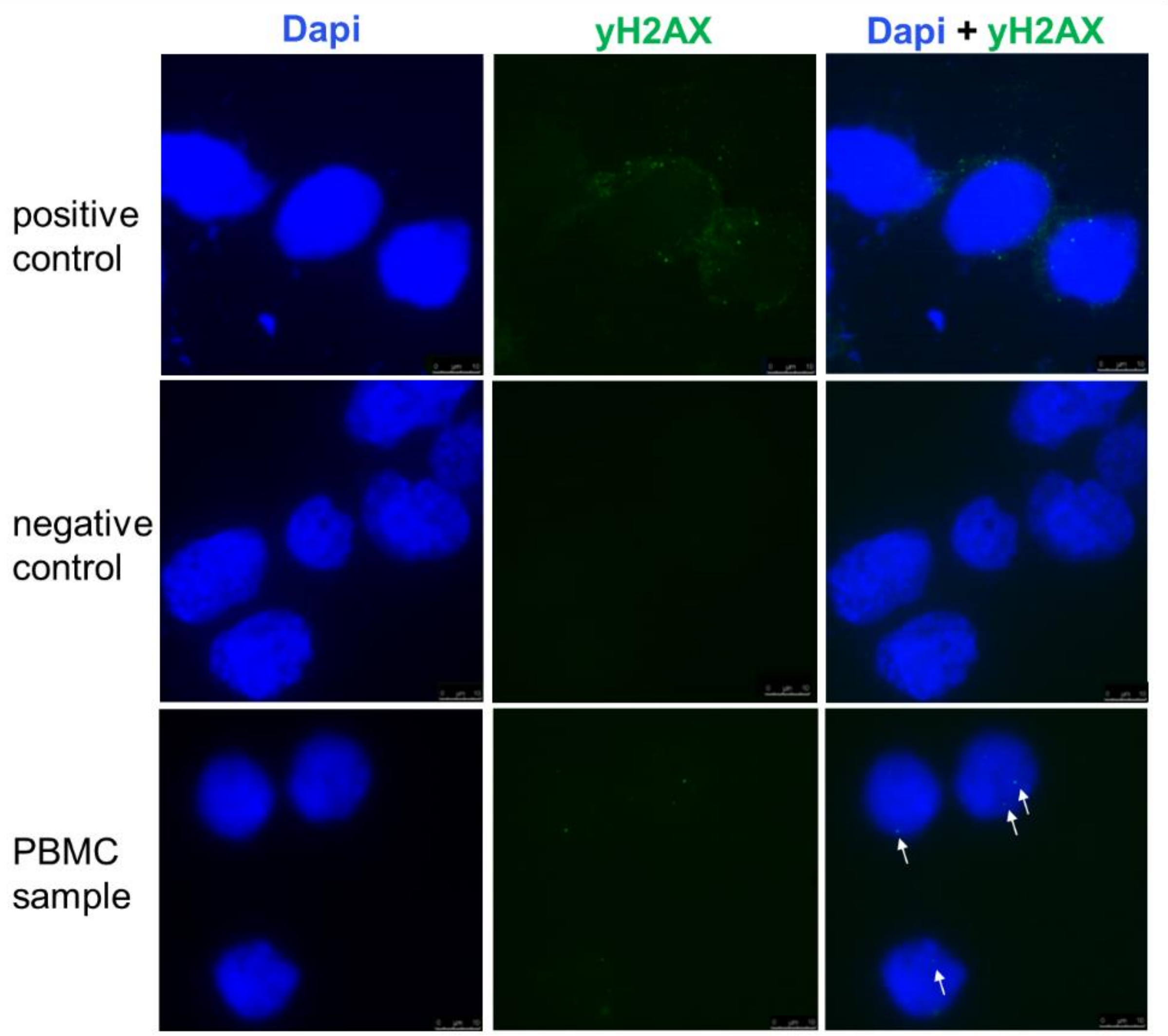

Supplementary Figure S.4: Exemplary representation of γH2AX fluorescence staining of a positive control (Hela cells, irradiated with X-ray), negative control (non-irradiated Hela cells) and PBMC of one study participant. Irradiated Hela cells show a high amount of γH2AX foci (green dots), whereas only few γH2AX foci are seen in PBMC and no γH2AX foci in non-irradiated Hela cells. Cell nuclei were counterstained with DAPI (blue). White arrows highlight γH2AX foci in PBMC.
DAPI = 4′,6-Diamidin-2-phenylindol; PBMC = peripheral blood mononuclear cells.

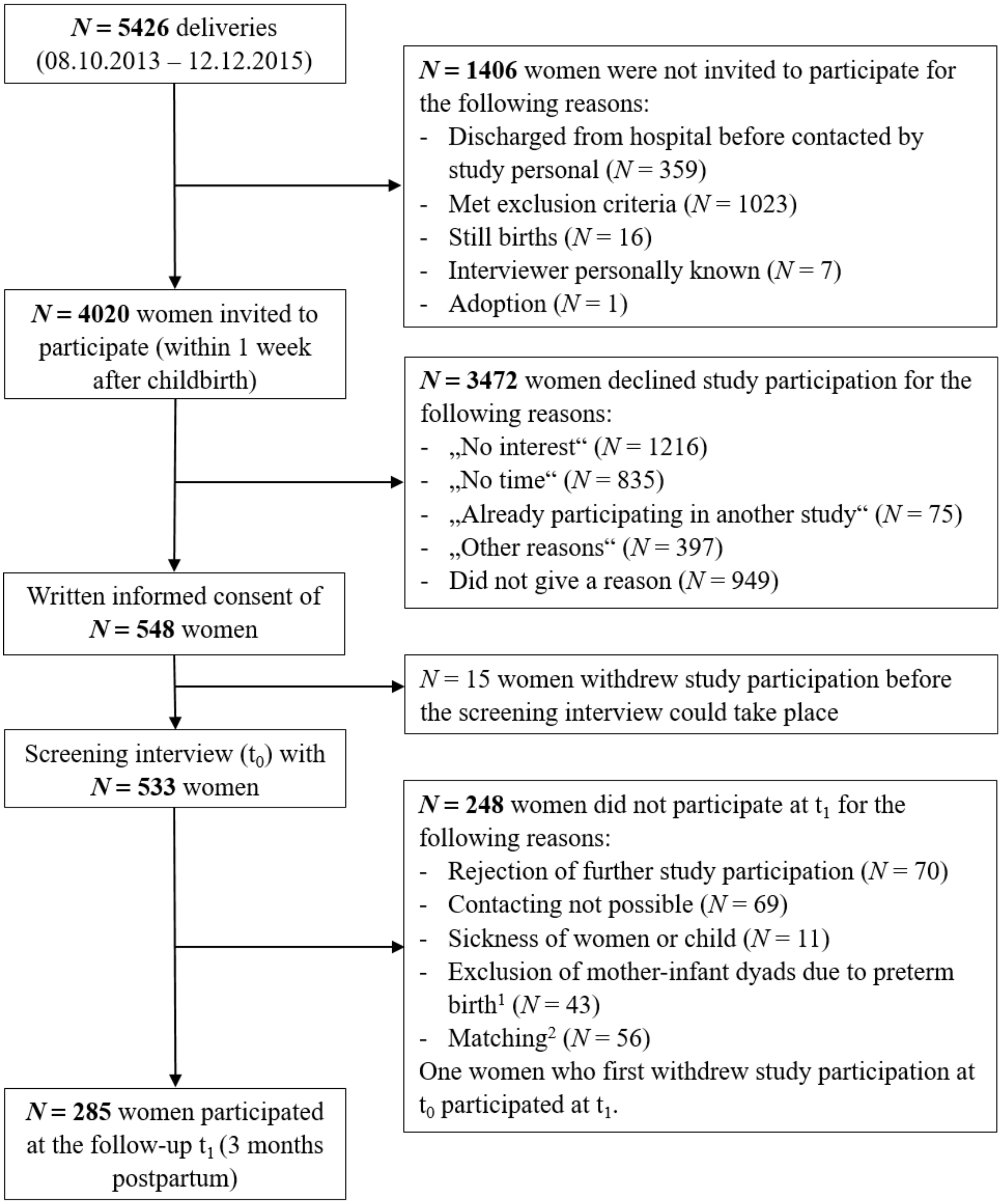

[1] Another main focus of the overall project included hypotheses investigating the effects of maternal childhood maltreatment experiences on the physiological development of the child. To this end, preterm birth was set as an additional exclusion criterion for t_1. [2] Using established cut-off criteria (see Bernstein & Fink, 1998), all women were categorized at t_0 into two groups: 1) women without any childhood maltreatment (CM) experiences (CM-) and 2) women with at least mild to severe CM experiences (CM+). All CM+ women were invited to participate at t_1. CM- women were matched to the CM+ women participating at t_1 according to the maternal age and their socioeconomic status. *N* = 56 CM- women were not invited for t_1 due to this matching process.

Supplementary Figure S.5: Study design of study cohort II as part of the project “My Childhood – Your Childhood” with drop-out rates.

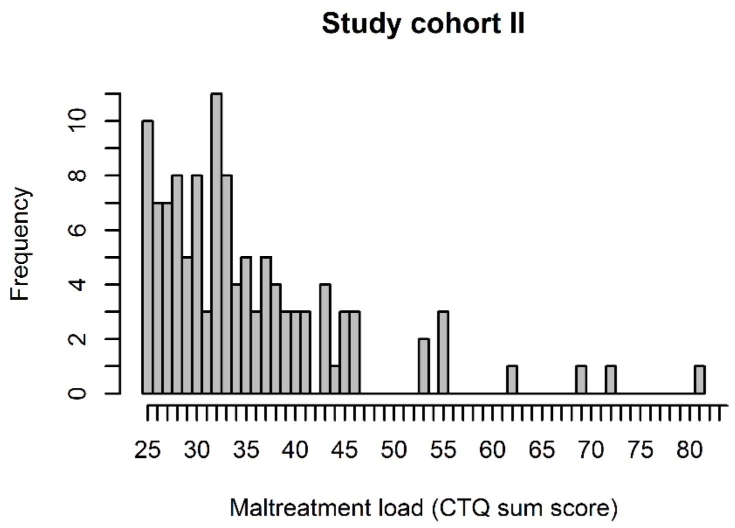

Supplementary Figure S.6: Distribution of the maltreatment load in study cohort II (*N* = 117). CTQ = *Childhood Trauma Questionnaire*.

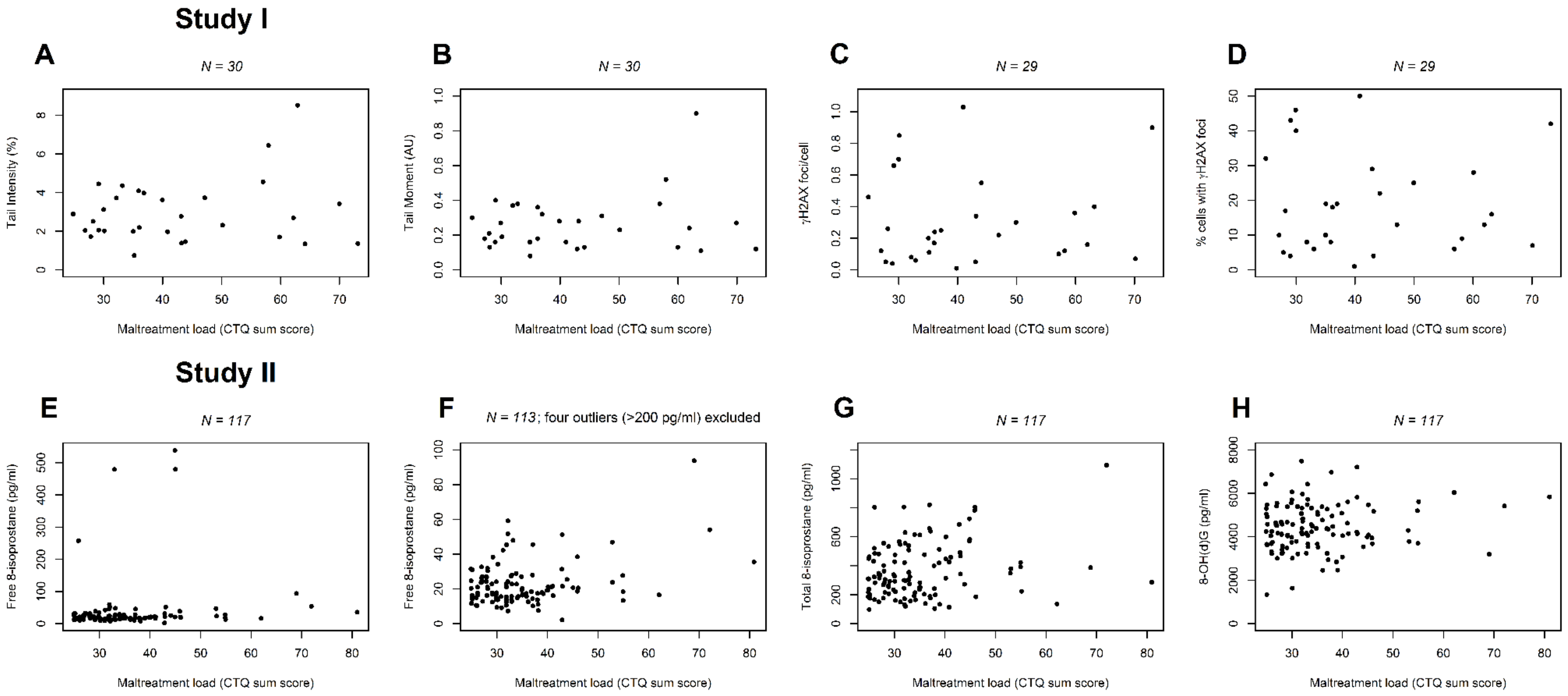

Supplementary Figure S.7: Scatterplots for the graphical illustration of the raw data used for statistical analyses: Oxidative stress biomarkers in study cohort I (A-D) and study cohort II (E-H) were plotted against the maltreatment load (CTQ sum score). In study cohort I, cellular oxidative DNA damage in PBMC was measured by tail intensity (percentage of DNA in the tail; A) and tail moment (tail intensity x tail length; B) of the Comet Assay (N = 30). As outcome variables of the γH2AX fluorescence staining, the number of γH2AX foci per cell (γH2AX foci/cell; C) and the percentage of cells with γH2AX foci (D) were analyzed in N = 29 women. In study cohort II, free 8-isoprostane (E, F) and total 8-isoprostane (G) in plasma were measured as markers for lipid peroxidation, 8-OH(d)G (H) in serum as marker for DNA damage (N = 117). For free 8-isoprostane, four outliers (>200 pg/ml) were excluded in a second analysis step (F).
AU = arbitrary unit; CTQ = *Childhood Trauma Questionnaire*; PBMC = peripheral blood mononuclear cells; 8-OH(d)G = 8-hydroxy-2'-deoxyguanosine and 8-hydroxyguanosine.
